# Suitability Analysis and Projected Climate Change Impact on Banana and Coffee Production Zones in Nepal

**DOI:** 10.1371/journal.pone.0163916

**Published:** 2016-09-30

**Authors:** Sailesh Ranjitkar, Nani M. Sujakhu, Juerg Merz, Roeland Kindt, Jianchu Xu, Mir A. Matin, Mostafa Ali, Robert J. Zomer

**Affiliations:** 1 Key Laboratory of Plant Diversity and Biogeography of East Asia, Kunming Institute of Botany, Kunming 650201, China; 2 World Agroforestry Centre East and Central Asia, Kunming 650201, China; 3 HELVETAS Swiss Intercooperation, Lalitpur 44700, Nepal; 4 World Agroforestry Centre, United Nations Avenue, Gigiri, 30677, Nairobi, 00100, Kenya; 5 International Centre for Integrated Mountain Development, Lalitpur 44700, Nepal; Monash University, AUSTRALIA

## Abstract

The Government of Nepal has identified opportunities in agricultural commercialization, responding to a growing internal demand and expansion of export markets to reduce the immense trade deficit. Several cash crops, including coffee and bananas, have been identified in the recently approved Agriculture Development Strategy. Both of these crops have encouraged smallholder farmers to convert their subsistence farming practices to more commercial cultivation. Identification of suitable agro-ecological zones and understanding climate-related issues are important for improved production and livelihoods of smallholder farmers. Here, the suitability of coffee and banana crops is analyzed for different agro-ecological zones represented by Global Environmental Stratification (GEnS). Future shifts in these suitability zones are also predicted. Plantation sites in Nepal were geo-referenced and used as input in species distribution modelling. The multi-model ensemble model suggests that climate change will reduce the suitable growing area for coffee by about 72% across the selected emission scenarios from now to 2050. Impacts are low for banana growing, with a reduction in suitability by about 16% by 2050. Bananas show a lot of potential for playing an important role in Nepal as a sustainable crop in the context of climate change, as this study indicates that the amount of area suited to banana growing will grow by 40% by 2050. Based on our analysis we recommend possible new locations for coffee plantations and one method for mitigating climate change-related problems on existing plantations. These findings are expected to support planning and policy dialogue for mitigation and support better informed and scientifically based decision-making relating to these two crops.

## Introduction

In Nepal, agriculture is a major source of income and forms the basis of livelihoods for the majority of the population. The importance of this sector for the nation’s economy was underlined with the approval of a revised Agriculture Development Strategy (ADS) in 2014, which supports further commercialization of the sector [1]. The ADS will guide the agricultural sector of Nepal over the next 20 years and one of it’s most important aspects is strengthening agribusiness. The strategy supports a shift from pure subsistence to a more commercial agriculture sector with the expansion of cash crop production, increased produce exports and import substitution. Two crops, banana (*Musa* spp) and coffee (*Coffea arabica*), both saw a huge expansion over the last decade concerning plantation area and production. Wild *Musa* species are native to Nepal [2], although the history of growing bananas in plantations is not clear. Domestication of bananas has been recorded for over a thousand years in the Ganges plains and adjoining regions [3], which suggest that banana plantations in the lowlands of Nepal are unlikely to be a recent development. Coffee was introduced to Nepal in 1938 but took a long time to be established in commercial plantations. The first commercial plantation in Nepal was established in 1981, after which plantation growing spread to 41 out of 75 districts [4,5]. Rapidly expanding tourism in Nepal after 1990 led to increased consumption of coffee in Nepal, while at the same time coffee drinking became increasingly popular among Nepalese themselves, resulting in an expanding domestic market. According to information from the Ministry of Agriculture Development [6], the plantation area of both crops are rapidly expanding. In 2014, domestic production only met about 90% of total domestic demand for bananas. Coffee exports from Nepal continue to increase, and it is now established as one of the country’s most important agricultural commodities [6,7].

For both crops, increasing production is an important goal—not only through expansion of plantation area, but also through increased productivity. This would enable the Nepali coffee and banana sectors to both meet growing demand and to improve the economic condition of smallholder farmers and entrepreneurs engaged in these two value chains. At the same time, there is a need to deal with issues related to land security issues and climate change [8,9]. Climate change will have greatly impact subsistence or smallholder farmers and the poor [10,11], while large-scale commercial plantations and associated agribusiness will be similarly affected [12]. Climate change is expected to affect agriculture crop production throughout the greater Himalayan region including Nepal. Climate variability has affected livelihoods and food security in Nepal in the past [13,14], and these impacts are predicted to increase in the future. The Coupled Model Inter-comparison Project Phase-5 (CMIP5) simulations and its scenarios indicate that by the mid-21^st^ century, mean annual temperatures will exceed 1.6–2.5°C above the late-20^th^-century baseline in Nepal [15]. Precipitation projections correspond to observed historical trends, which suggest decreases in post-monsoon rainfall in the winter months, potentially leading to drought conditions [14]. These changes are expected to have significant impacts on crop production with varying impacts depending on elevation, crop type, and growing season [16]. For example, climate change is likely to drive geographic shifts in crop and land suitability, result in changes in the occurrence of crop diseases and pests [17], and directly impact yields of the main crop species [18–20] and varieties. These impacts may pose a major threat to different cash crops in the near future. Commodities like banana and coffee are not an exception to the potential impacts of climate change; it is probable that climate change impacts may be significant in monoculture plantations, which could be more susceptible to climatic and biological hazards [21]. If the potential effects of climate change are not addressed through appropriate changes in farming practices and techniques then decreasing yields will impact both local and national food security [22] and trade relations. Adaptations to climate change in terms of farming practice and techniques could include measures such as moving cultivation to different areas and elevations, taking advantage of advances in agricultural technology such as abiotic-stress resilient crops [23,24], or other improved management practices such as agroforestry and integrated pest management [13,25,26].

Selection of the proper crop variety for the proper geographical location, taking into account the potential impacts of climate change [27], is essential if the rural economy and livelihoods of smallholder farmers in Nepal is to anticipate and successfully adapt to projected climate change impacts. Key factors for proper crop selection include sustenance, market dynamics, productivity, cultural preferences, organizational roles, and weather conditions [13,27–29]. In developing countries like Nepal, these factors play a decisive role—in particular, market dynamics alone can drive changes in cropping systems. Different governmental and non-governmental organizations (e.g. HELVETAS Nepal) are promoting cash crops such as banana and coffee. Such activities aim to build the capacity of farming families to cope with changes by teaching them to grow new crops and/or use improved growing techniques (e.g. [4]). Once the crops are planted and investments are made, however, the household is to a large degree locked in to the cultivation of the new crops. Therefore, it is important to develop high-value, perennial cropping systems, and identifying suitable climatic zones is an important preparatory step in this effort.

Adaptation of agricultural systems to climate change can potentially be addressed through a number of strategies including the spatial shifting of crop production systems to follow suitable climatic conditions, conversion of monoculture plantations to intercropping systems [9,30,31], or the application of improved production practices. It is also essential to develop models that can be used to predict how the locations and extents of areas suitable for the production of focal crop species will change under future climate change scenarios. Likewise, projected warmer and wetter bioclimatic conditions [15] and the shift of bioclimatic zones upslope [32,33] may present opportunities for crop diversification, open new areas for plantations, as well as increasing the potential for future expansion of these cropping systems. Species distribution modelling (SDM) can be an inexpensive, quick and flexible tool for identifying the climatic envelope and projections of climate impacts on the crops [12,34,35]. Identifying suitable climatic zones before planning crop plantations can greatly minimize input costs such as externally supplied heat and irrigation [12]. In addition, SDM can be used to match adaptation policies and practices to anticipated or observed climate change [36]. An ensemble approach which combines various modelling algorithms based on their individual performance and generates a consensus map greatly reduces the uncertainties which would result from the use of a single model [37]. BiodiversityR provide sets of functions for suitability mapping based on an ensemble of modelling algorithms. The advantage of this package is that it allows direct handling of model formulae and the functions that select algorithms for ensemble output [38]. The ensemble is based on consensus results that combined only the most successful algorithms, according to the statistical criterion [38,39]. In this study, we analyze and model current and projected future bioclimatic conditions to assess the suitability and spatial delineation of coffee and banana production zones, the impacts of climate change, potential new production zones, and intercropping opportunities for selected crops.

## Materials and Methods

### Study sites

**Fig 1:**
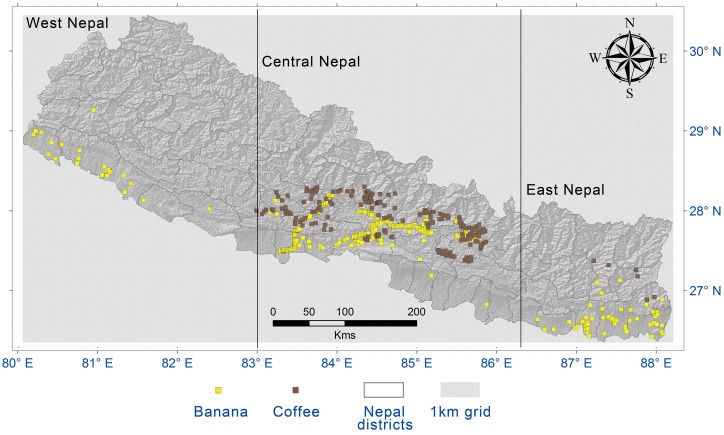
Banana and coffee plantation locations in Nepal.

Nepal, with an area of 147,181 km^2^, is characterized by highly heterogeneous terrain and fragile mountain environments with high elevations (8,848 m asl) in the northern Himalayas and low elevations (<70 m asl) in the southern plains. Based on phytogeography, Nepal is divided into west (west to 83°), central and east (east to 86.3°) Nepal (Fig 1) [40]. In the summer monsoon, east Nepal receives higher rainfall than west Nepal, while winter rains are more common in the western part of the country [41]. Nepal hosts a wide diversity of climatic zones and habitat types, ranging from tropical to alpine, as well as a lifeless nival zone [42]. About 17% of plains land in the lower belt is covered by plantations of a wide variety of tropical crops. The remaining part of the country is a hilly and high mountain region, where the climate is sub-tropical to alpine and suitable for a different range of crops. Bananas grown on commercial plantations require mean annual temperatures in the range of 26–30°C and annual rainfall of 2,000 mm or higher [43], which occurs in the lowland areas and some inner valleys of Nepal. The optimal mean temperature for coffee is about 22°C, with rainfall between 1,400–2,000mm [44], which occurs in the hilly regions of Nepal.

### Crop data

We compiled secondary data from yearly statistical reports, district profiles, newspapers and online sources, and reports from different organizations [e.g. 5] (see S1 References). The secondary data provide important information on commercial plantation districts and the extent and location of banana and coffee growing (S1 Dataset). We visited several districts and localities during 2014 and recorded plantation locations. Another source of plantation information was interviews with smallholder farmers, community-based organization staff and field-based staff of HELVETAS Nepal. Based on the dataset compiled we prepared a grid for the presence of two crops (Fig 1). We conducted grid-based analysis using 30 arc second resolution bioclimatic and geo-data set to generate production zone mapping. A high number (n = 10,000) of background (pseudo-absence data) was selected within a diameter of 200km around geo-referenced crop points. All the points that were outside the areas of interest were discarded. We performed Moran’s I test to check spatial auto-correlation after data treatment. Moran’s I values were 0.5 and 0.4 (p<0.001) for banana and coffee, respectively, indicating clustering of the data [45] and the need for further treatments (see the section below on Ensemble model and evaluation).

### Predictor variables

Following Metzger et al. [46] four bioclimatic layers were used as the input to the ISODATA clustering routine in ArcGIS (10.1) to classify the Global Environmental Stratification Strata (GEnS). These layers are: temperature degree day, aridity index, mean monthly temperature seasonality and potential evapo-transpiration seasonality. GEnS is a statistical stratification of the world’s land surface into 125 homogeneous bioclimatic strata, representing a considerable advance over earlier attempts at ecosystems mapping [33]. These strata have been aggregated into 18 global environmental zones (GEnZ) which cluster together areas with similar climates [32,46]. GEnS can quantitatively relate spatial distribution and estimate the direction and magnitude of impacts on ecosystems and crops production zones. It provides a consistent methodology across landscapes that have so far mostly been studied using a mixture of different protocols and approaches [33]. The statistical signature profiles of the strata have been reconstructed for Nepal based on a multivariate analysis (maximum likelihood classification) of these four variables. We identified 47finer resolution GEnS within the political boundary of Nepal, which were aggregated to 11 of the Global Environmental Stratification Zones (GEnZ, see S1 Table) following Metzger et al. [46].

**Fig 2:**
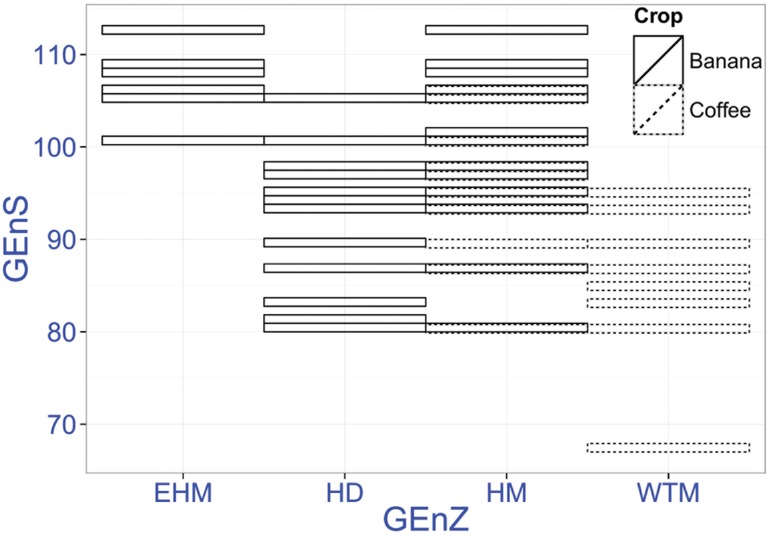
Bioclimatic strata (GEnS), within the major bioclimatic zones (GEnZ) at the cultivation areas of banana and coffee in Nepal. Overlap of color in the strata represents occurrence of both crops (EHM—extremely hot and moist; HD—hot and dry; HM—hot and mesic; WTM—warm temperate and mesic). GEnS is represented numerically (nominally arranged along an increasing average annual temperature gradient) in a range from 1–125. Banana and coffee plantations were found between strata 66–113.

The GEnZ of the region were used to identify various levels of optimum spatially delineated bioclimatic conditions for coffee and banana production throughout Nepal. We found three GEnZ, comprising 18 GEnS that were suitable for banana while 17 GEnS in two GEnZ were identified as suitable for coffee (Fig 2). Banana was widely represented in different GEnS while coffee was found in small patches of many strata.

Zomer et al. [32] used this modeled environmental stratification approach to examine the impact of projected climate change on eco-regions within the Kailash Sacred Landscape and on rubber plantation areas in Xishuangbanna, China [33]. We used this approach, along with other bio-climatic variables obtained from different sources (S2 Table) to examine the potential distribution of banana and coffee crops using multi-model ensemble modelling based on the theory of ecological niche modelling (ENM). The bioclimatic variables were selected based on iterative calculations of variance inflation factors (VIF) [39,45,47], where VIF with >10 were eliminated (S3 Table) to list a set of least correlated bioclimatic variables. The bioclimatic variables including GEnS, physical variables such as aspect, slope and land-cover (LC) were used to calibrate and generate the ensemble model (Table 1). LC [48] was treated as a dummy variable and was used as a limiting factor in the distribution model (detailed category of LC is shown in S4 Table).

**Table 1:** Bioclimatic and geographical data set used to predict suitability of two crops.

*Descriptors*	*Name of variable*	*Unit*	*Crop(s)*	*Range*	*VIF score*
*Area*	Nepal				
*Years*	1950–2000 as baseline/2050 as future				
*Changing*	**AI** = Annual aridity index	×10000	Banana	>0.65 (humid)	8.49
			Coffee	>0.5 (sub-humid- humid)	3.86
	**bio_2** = Mean Diurnal temperature	°C×10	Banana	6 to 7°C	2.40
			Coffee	9.5 to 12.5°C	2.46
	**bio_3** = Isothermality	percent	Banana	45 to 48	2.82
			Coffee	43 to 46	2.36
	**bio_14** = Precipitation of Driest month	mm	Banana	5 to 8mm	2.25
			Coffee	10 to 12mm	1.73
	**bio_15** = Precipitation seasonality	percent	Banana	<60	7.32
			Coffee	95	6.38
	**bio_18** = Precipitation of Warmest Quarter	mm	Banana	1000 to 1500mm	10.18
	**bio_19** = Precipitation of Coldest Quarter	mm	Coffee	130 to 160mm	3.98
	**pet_sum** = Potential evapo-transpiration for summer months	mm/month	Banana	>150mm	4.69
			Coffee	120 to 150mm	2.66
	**GEnS** = Global Environmental Stratification	strata	Banana		
			Coffee		
*Unchanging*	**ASP** = Aspect	direction	Coffee	East to Southwest	
	**Slo** = Slope	degree	Banana	<12	
			Coffee	15	
	**LULC** = Land use land cover	dummy	Banana		
			Coffee		

Source: www.worldclim.org; www.csi.cgiar.org; www.eros.usgs.gov; www.icimod.org.

### Ensemble model and evaluation

We used BiodiversityR package (version 2.4–1) in R [38] to prepare for consensus mapping of species distributions. Consensus mapping is a mapping technique that is based on an ensemble of several niche-modelling algorithms (sub-models). We calibrated 19 ENM algorithms (S5 Table) and the best-fitted sub-models were selected for an ensemble output. Following Hijmans [49] and Ranjitkar et al. [45,50], we used 4-fold cross-validation, where crop location and background data was partitioned into 75% calibration and 25% evaluation. Spatial autocorrelation among species presence and background points was quite high, as indicated by the Moran I test (see section Crop data). We evaluated the ability of sub-models to cope with spatial autocorrelation by calculating calibrated Area Under the Receiver Operator Curve (cAUC) values and comparing these with a geographical null model [47].

Using Hijmans’ method for spatial sorting [49], bias was removed through several rounds of calibrating and evaluating all models (including the geographical null model), each time using three partitions for model calibration and one partition for model evaluation [39,45]. Details of sub-models and cAUCs are provided in the Supporting Information (S6 Table). We repeated this process and compared the 20 resulting cAUCs of each ensemble models with that of the geographical null model by means of Mann-Whitney tests. Removal of spatial sorting bias in testing data for different model calibrations yielded AUC values for the null model between 0.48 and 0.51 (for both crops), which is equivalent to a random draw [49], and cAUCs values of the different individual modelling algorithms between 0.55 to 0.80 for both crops (significantly different from null model; Mann-Whitney tests, p < 0.05 in all cases). Only sub-models that gave cAUC values, which were significantly higher than the null model were retained in the ensemble model used for projections.

The cAUC values of the sub-models were used to determine the appropriate weights (range between lowest 0 to highest 1) for the ensemble model [38]. A second calibration was applied where modelling algorithms with weights >0.05 were retained. The second calibration use 10 internal test runs and 4-fold cross-validations resulting in final weights for the selected algorithms, which were used to produce the final ensemble output. The tuning process in the software explored how differences in weights of the sub-models resulted in changes in the cAUC of the ensemble model, and selected the weights that result in the greatest accuracy of the ensemble model [45]. For each focal crop, calibration was repeated multiple times, resulting in conclusive average weights for modelling algorithms. The ensemble model (*P*_*ensemble*_) was calculated using the following formula described in Ranjitkar et al [50]:

$$P_{ensemble}=\;\frac{\sum \left(w_{mod}P_{mod}\right)}{\sum w_{mod}}$$

where, *w*_*mod*_ = weighted averages of sub-models (*P*_*mod*_)

The ensemble that yielded the highest sum of cAUC values was considered the most appropriate scenario for projecting to future climate conditions, respectively.

### Future projections

First, the GEnS up to 2050 were reconstructed by an ensemble of 19 Earth System Models provided by the 19 Coupled Model Intercomparison Project—Phase 5 (CIMP5; [51]), using the same set of significant variables described above for GEnS. Within each of the 19 CIMP5, there are four representative concentration pathways (RCP) [52], ranging from RCP 2.6 (aggressive mitigation / lowest emissions) to RCP 8.5 (highest emissions scenario). All models available within each RCP were combined into a majority ensemble result, using the class with the majority of occurrence within any particular grid cell as the class for that location [53]. Mora et al. [54] tested the robustness of the CIMP5 model ensemble based on historical observation data (1985–2005) and found a high correlation when using multi-model averages.

Reconstructed GEnS and bioclimatic layers were prepared based on the ensemble CIMP5 projections. The layers were used to model the future spatial distribution of climatic suitability for coffee and banana crops. Aspect and slope were used as limiting factors. LULC classes (e.g. agricultural land, built-up area, forested area; see S3 Table), the future projections of which have not yet been created, were used as a dummy variable in all models. Land use change is likely to occur within the next 30–40 years due to the expansion of populated areas, cultivation of new land in some areas, and reforestation in others; however, for the purposes of this study and the limits of the available data, we have had to assume that land cover remains stable.

The next step was projecting into future climatic scenarios. The calibrations and weights determined as described above were projected into ensemble results of each RCPs as future climatic layers to generate ensemble outputs that included consensus raster layers for future climatic scenarios. The consensus layer summarized the presence-absence (1–0) of focal species for each grid cell based on a threshold defined by maximizing the sum of the true presence and true absence rates.

### Predicting potential zones for mixed plantations

All pixels in the consensus map output were classified according to the cut-off point, based on the threshold as mentioned above. A score of above this threshold represents the suitable climatic space for the focal species [39,45], such that all pixels with suitability scores above the cut-off point were included in the final map representing species’ bioclimatic space for focal species. The future projections were averaged across each RPC scenario and for each crop. The future projection for banana crops was overlaid with the current suitable layer of coffee to identify overlapping regions, which indicated possible intercropping zones where banana can be used as a shade tree for coffee bushes. This information was used for recommending locations for possible mixed plantations. A fuzzy logic model was employed for identifying overlap of climatic spaces of the two focal species, and areas with potential for mixed plantations of these two crops were identified.

## Results

### Assessment of predictors

Bioclimatic assessment of the two focal crops was presented in Table 1. Results from the model show a comparatively narrower “mean diurnal range” (bio2) for banana and a comparatively wider range for coffee. The results indicated that for banana cultivation, the difference between the maximum and minimum monthly mean temperature should be less than that for coffee. Isothermality (bio3) value for both crops was between 43–48%, indicating a smaller level of temperature variability within an average month relative to the entire year. For both crops, at least 10mm of precipitation was required during the driest month of the year (bio14). However, banana suitability occurred in areas with a substantially lower (90) precipitation seasonality value (bio15), and coffee was found at significantly higher precipitation seasonality values (90 to 120). Precipitation in the warmest quarter of the year (bio18) ranging from 1,000 to 1,500mm was found necessary for banana suitability. About 50 to 80mm of precipitation in the coldest quarter of the year (bio19) was necessary for coffee cultivation. Aridity index (AI) indicated that humid conditions were required for banana growing, while semi-humid conditions were optimum for coffee plantations. For bananas, potential evapo-transpiration during the summer months (pet_sum) of more than 150mm was optimum, while the most desirable conditions for coffee were around 120 to 150mm. Flat areas and slopes of less than 12 degree inclines were suitable for banana growing, while for coffee slopes of around 15-degree inclines were optimum (Table 1). Results based on the landuse layer showed that both crops are mostly currently confined to agricultural land, while climatic factors in bare land and grassland areas were found to be suitable for both crops. The edge of broadleaf open forest was found to be a particularly suitable location for coffee, while other landuse types were found to have limiting effects on the plantation of both crops. Aspect was used as a predictive factor for coffee production, but it was not shown to be a crucial factor.

### Suitability modelling

The trained and tested models were applied using the geospatial dataset for the selected bioclimatic variables, geographical, landuse and environmental stratification (listed in Table 1), to give a distribution map of current suitability for each of the two crops. Results from models with positive weights were combined into ensemble outputs that included consensus spatial layers for current climatic conditions. In the final ensemble output, 15 sub-models were used for banana and 15 sub-models for coffee (see S7 Table). All the habitat suitability sub-models for banana show consistency and used in the final output of results. In the case of coffee, habitat suitability for coffee was consistent in most of the models.

**Fig 3:**
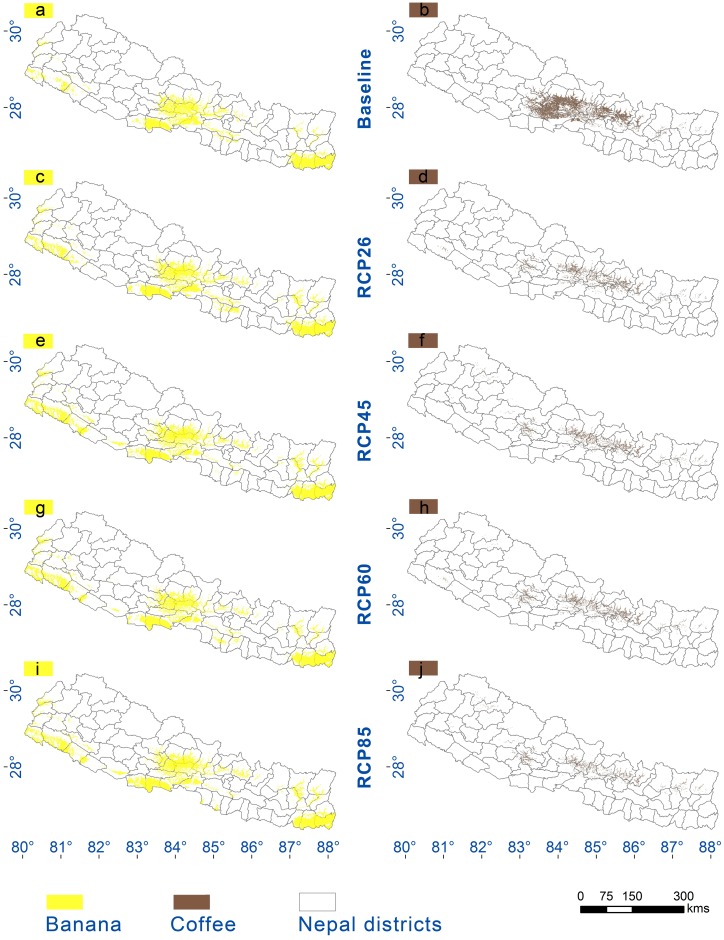
Banana and coffee suitability zones based on ensemble output for current conditions (a and b) and for future (c-j). Banana is represented by yellow colouring and coffee by brown.

A final suitability map (Fig 3a and 3b) was prepared based on the ensemble outputs, which showed that suitability zones for banana occurred mostly in the lowland Terai region from West to East Nepal while coffee suitability occurred in the mid-hill region in central Nepal. Dense forest areas fragmented continuous climatic suitability of the crops. Agricultural land provides most of the suitable area for both crops at present. Also, suitability for both crops was detected close to the edge of the forest (broad-leaved and needle-leaved) and was a suitable location for plantations. Approximately 9% land area of Nepal and about 25% of the agricultural landscape was shown to be suitable for bananas under current climatic conditions, mainly in lowland areas. Likewise, about 7% of Nepal and 11% of the agricultural landscape was found to be suitable for coffee, mostly in the mid-hills between 600 and 1,350 m asl.

### Future assessment

**Fig 4:**
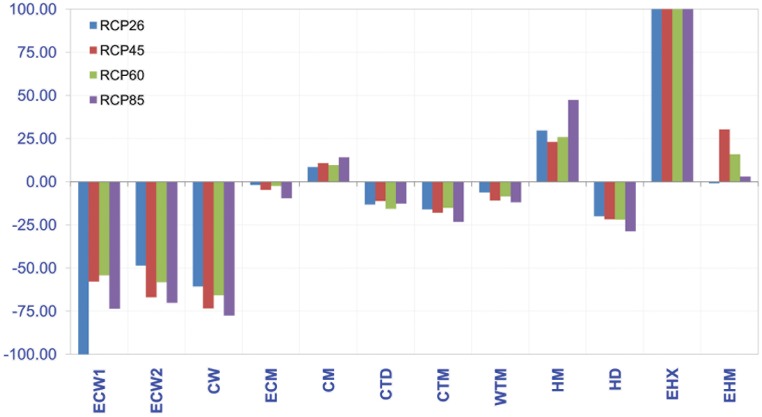
Change in GEnZ in Nepal across four CIMP5 RCP scenarios compared to current conditions. (CM—cold and mesic; CW—cold and wet; CTD—cool, temperate, and dry; CTM—cool, temperate, and mesic; ECM—extremely cold and mesic; ECW—extremely cold and wet; EHM—extremely hot and moist; HD—hot and dry; HM—hot and mesic; WTM—warm temperate and mesic; y-axis stands for percentage change).

Substantial changes in the spatial distribution of bioclimatic zones and their associated strata were evident in the projected future distribution maps—for instance, the colder zones such as ‘extremely cold and wet’ and ‘cold and wet’ were reduced by about 60–70%. The onset of completely novel bioclimatic conditions was evident through the appearance of the ‘extremely hot and xeric’ zone (Fig 4), a set of bioclimatic conditions not currently seen in present-day Nepal. In addition, noticeable changes indicated that there will be significant displacement of agro-ecological zones which will ultimately impact crop production and food security. For the two focal crops, changes in relevant bioclimatic zones, notably ‘Hot and mesic’ (expansion), ‘Hot and dry’ (reduction), ‘Extremely hot and moist’ (expansion), and ‘Warm temperate and mesic’ (reduction) will have direct impacts. In addition to that, all the CIMP5 scenarios show the appearance of this new “extremely hot and xeric” zone in the lowlands in Nepal (see Fig 4).

Changes in the suitable area available for the two focal crops by 2050 were indicated by changes in suitability scores averaged across all four RCPs. The current suitable area for banana was projected to decrease by 16.7±2.1% by 2050. The current suitable area for coffee was projected to decrease by 72.6±4.4% by 2050. About 40.7±5.2% new area will become suitable for banana plantations, but only 11.9±2.3% of new suitable area for coffee (Table 2). The final ensemble output indicated that climatic conditions would improve for banana cultivation in the future, while conditions for coffee will become worse.

**Table 2:** Results of the ensemble models showing changes in suitable and non-suitable areas, including area that lost suitability and new potential area for plantation of selected crops by 2050.

Crop	Scenario	Pixel count in the ensemble raster layers					In percentage		
		Unsuitable	Suitable	Lost	Gain	Total	Suitable	Lost	Gain
Banana	Baseline	175456	17678			193134	9.15		
	RCP26	169329	15348	2330	6127	193134	7.95	13.18	34.66
	RCP45	167578	14698	2980	7878	193134	7.61	16.86	44.56
	RCP60	168998	14396	3282	6458	193134	7.45	18.57	36.53
	RCP85	167157	14467	3211	8299	193134	7.49	18.16	46.95
	Average							16.69	40.67
Coffee	Baseline	179339	13795			193134	7.14		
	RCP26	178216	4481	9314	1123	193134	2.32	67.52	8.14
	RCP45	177571	3763	10032	1768	193134	1.95	72.72	12.82
	RCP60	177587	4056	9739	1752	193134	2.10	70.60	12.70
	RCP85	177373	2836	10959	1966	193134	1.47	79.44	14.25
	Average							72.57	11.98

The baseline represents the current scenario, while RCPs represent future scenario by 2050 (2040–2060); pixel count is total number of cell in the raster layer within study area (calculated in BiodiversityR package) that represent suitable and unsuitable area for the crops; percent suitability = (suitable pixel×100)/Total pixel; percent loss/gain = (lost/gain pixel×100)/suitable pixel.

**Fig 5:**
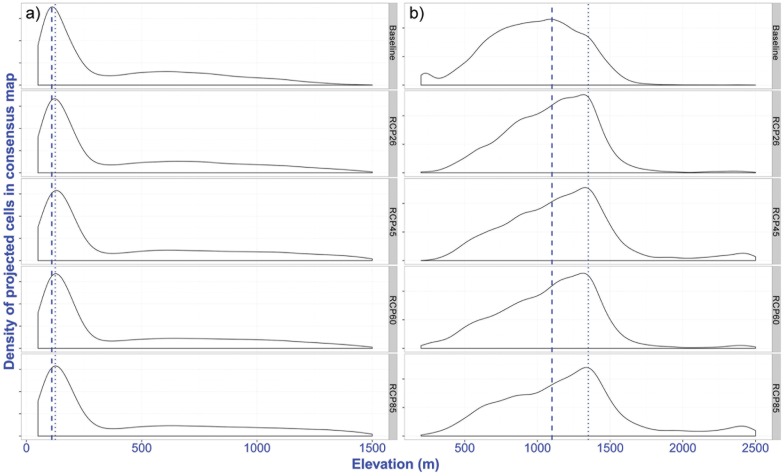
Change in major production zones for a) banana and b) coffee in Nepal across four RCP scenarios compared to current conditions. The dashed line indicates highest density of cells representing maximum suitable area in the baseline scenario and a dotted line indicates changes in suitable area and a shift in elevation in future scenarios compared to current suitable elevation. All the layers generated for baseline and future scenarios are available at http://landscapeportal.org/layers/.

The models show suitable areas for banana mostly below 1,200m in the baseline scenario, with the major production zone occurring below 300m (Fig 5a). By 2050, the limit of suitable area was predicted to shift upwards to about 1,500m (Fig 5a RCP scenarios). The upward mean elevation shift in the major production zones was found to be about 15m by 2050. Likewise, the area suitable for coffee cultivation remained below 1,500m. Between 600 m to 1,350m, a major shift was indicated, with major production zones shifting by about 150m by 2050 (Fig 5b). Projections across different scenarios showed suitability might occur up to about 2,500m by 2050 (Fig 5b RCP scenarios). Fuzzy overlay of the future suitability of banana and current suitability of coffee revealed that in 37.3±2.0% of current coffee suitable areas, banana can be introduced for intercropping. Suitability for intercropping mostly occurred between 700 to 1,200m elevation.

## Discussion

Computer modelling has been a feature of agricultural research for decades and the application of SDM has been increasing in the prediction of future suitability distribution of important cash crops like banana and coffee [12,34,55]. SDMs are gaining popularity in crop modelling because it saves a lot of time, energy and money rather than trying out each possible set of actions through trial and error [12,35]. Reliability of such models can be improved by minimizing the prediction uncertainties and ensemble modelling is one measure to minimize uncertainties due to single model prediction [37,45,56].

Our results indicate that significant expansion of suitable areas for tropical crop bananas will occur with rising temperatures. This expansion will be accompanied by the appearance of novel (warmer) GEnZ. The comparison of current and future spatial distribution of bioclimatic conditions showed large and substantial shifts, also reported by Zomer et al. [32] in the Kailash region, where upward shifts of the mean average elevation of GEnZ by 357m and eco-regions by 371m were projected. In addition, they reported the expansion of lower tropical and sub-tropical zones and eco-regions similar to the current study. In ecological modelling, multivariate environmental similarity surface (MESS) analysis is used to detect novel climate across times and places [57], which is not sampled in the calibration data. In our study, comparison of GEnZ provides information on the novel bioclimatic zones. Therefore, we did not present MESS results.

Our results for banana crops show the positive changes in suitability of banana in the lowlands areas, including lower elevation areas in the middle hills. Banana plants have a short lifespan, and are relatively adaptable to future conditions. This indicates that banana could be a key crop in the future context of climate change, in which it will also be important to increase national production and improve the livelihoods of smallholder farmers. In contrast, the long lifespan of coffee—ranging from about 30 to 50 years—is likely to mean current plantations will struggle to adapt to the impacts of future climate, as the majority of the current coffee growing area in Nepal will become unsuitable in the future. Several research studies around the world have revealed coffee to be highly sensitive to climate change [34,55]. Throughout the world, a major decline in current coffee production zones has already been projected [55]. Current major coffee growing areas are predicted to suffer badly due to climate change, while there are predictions of a dramatic and profound decrease (13–90% under different modelling approaches) in the bioclimatically suitable localities of the world’s dominant coffee production regions (Brazil, Vietnam, Ethiopia, Sudan, and Kenya) by 2050 and 2080 [34,55].

In Nepal, the biggest factor responsible for a decline in the suitable production area of coffee by 2050 is the shift in bioclimatic zones in this region [32,53]. The predicted rise in temperature of 1.6 to 2.5°C, along with the tremendously steep slopes in the Himalayas is a major limiting factor in the suitability of coffee-producing areas. Research conducted in Uganda shows that rising temperatures are not only responsible for the reduction in the suitable production zones, but that they also reduce the size of coffee beans and the quality of coffee [58]. Therefore, climate change will likely have duel impacts on coffee production via shrinking production suitability zones and reducing the size of coffee beans produced.

Coffee in Nepal is still a curiosity crop for many farmers with little knowledge of proper plantation techniques, but an increasing number of smallholder farmers are attracted by this crop’s huge market potential. Although the national database [e.g. 5] shows continuous increases in the production and export of coffee, smallholder farmers continue to face cultivation related problems, including limited access to information on suitability based on climatic information, and uncertain future bioclimatic conditions. All these factors directly affect exports and the national economy as well as local communities. Therefore, we urgently need to develop improved practices or other mitigation measures to retain current plantation sites as well as production quality. In contrast, our model indicates that banana suitability zones will expand in the future, which offers the potential of boosting economic growth through increased banana cultivation. Our model however cannot predict the impacts of crop pests, soil conditions, and management-related issues that directly or indirectly affect yields. At present, crop pests are one of the major issues for both crops that farmers are struggling to deal with. To solve this problem, some farmers have practiced intercropping of different crops. Intercropping can increase total farm productivity and can bring other valuable benefits such as improvements in soil fertility and the suppression of pests and/or diseases [59,60].

Case studies in Uganda and Costa Rica shows that banana and coffee intercropping can benefit smallholder farmers, in addition direct effects on the crops themselves [58,60,61]. Even in Nepal, coffee-banana intercropping is reported beneficial than monoculture plantation [4]. According to the International Institute of Tropical Agriculture, coffee-banana intercropping is a climate-smart system where shade provided by banana can help coffee to cope with hotter climates and drought shocks. Research has shown that shade can reduce the temperature in the understory plants by up to 2°C or more [58]. Not only that, banana yield was reported to be higher in coffee-banana systems compared to mono-cropping, thus indicating that this technique can be economically profitable for coffee growers [60,61].

Our model revealed there was a possibility of intercropping coffee and banana. If banana can be intercropped with coffee, about 1/3^rd^ of current coffee-suitable zones and about 10,000 smallholder farmers could benefit. These smallholder farmers could receive economic benefits from increased productivity with limited land use. This could in turn mitigate pressure to expand farmlands and thus contribute to forest and biodiversity conservation. In addition to increasing farm productivity, intercropping systems can also contribute to minimizing pest problems and improving soil health. The effective implementation of this technique does require farmers to be trained and provided with proper information on the management and benefit of such intercropping.

## Conclusion

Spatial modelling of two cash crops successfully delineated the current bioclimatically suitable area for banana and coffee cultivation and the potential expansion of suitable areas by 2050. Future projections indicated that climatically-suitable areas for banana will expand in the future, indicating that future conditions will permit increased banana production. Regarding coffee, the future climate will likely not be as favorable. Huge areas of current plantation land will be lost in the future, most probably due to higher temperatures and shifting agro-ecological zones. Although climate change will open new areas to coffee plantations, particularly in the mid-western and far-western regions of Nepal, the question remains as to how to protect current production areas and farmers’ investments. In this study, we identified one possible measure of adapting to the impacts of climate change using agroforestry systems such as intercropping banana and coffee. We identified locations where coffee could be intercropped well with banana. African and Latin American highland experiences indicate that this kind of climate smart-system is beneficial for both crops and smallholder farmers [58,60,61]. In the Asian Highlands, some smallholder farmers are already benefitting from such agroforestry practices including Nepal [4]. The further promotion of these techniques could therefore help smallholder famers to adapt to climate change impacts while improving the productivity of their farms.

“The right crop in the right place” is imperative to ensure sustainable yields of the chosen crop and sustainable benefits to farmers. Our model provides a means of identifying suitable bioclimatic condition for crop plantations. Our results could be further confirmed and added to by ground observation that includes soil testing and an analysis of farmers’ current best options. Combining our results with ground testing can help developmental agents and policy makers to promote suitable cash crops in the right place. Along with this, market opportunities for smallholder farmers need to be properly addressed through cooperatives or other such local bodies, which secure adequate prices for farmers.

## Supporting Information

S1 Dataset (TXT)

S1 References References for crop data compilation. (DOC)

S1 Table Global Environmental Stratification of Nepal. (DOC)

S2 Table Bio-climatic and geophysical variables used in modelling process. (DOC)

S3 Table Variance Inflation Factor (VIF) in different test runs for selection of explanatory variables and correlation between predictor variables. (DOC)

S4 Table Land cover of Nepal. (DOC)

S5 Table Species distribution algorithms included in ensemble modeling in the BiodiversityR package and used in the present analysis. (DOC)

S6 Table cAUC for sub-models, null model and ensemble model in the different model calibration. (DOC)

S7 Table cAUC and weight. (DOC)
