# Supplementary material for: Suitability Analysis and Projected Climate Change Impact on Banana and Coffee Production Zones in Nepal
Source: PLoS One. 2016 Sep 30;11(9):e0163916. doi: 10.1371/journal.pone.0163916 (PMC5045210; doi:10.1371/journal.pone.0163916)
Supplement: S1 References — (DOC) [file pone.0163916.s002.doc]

**Supporting Information S1**

**References for crop data compilation**

1. GoV. 2013. National Sample Census of Agriculture, Nepal. Government of Nepal, National Planning Commission Secretariat Central Bureau of Statistics, Kathmandu, Nepal. 1 Taplejung
2. GoV. 2013. National Sample Census of Agriculture, Nepal. Government of Nepal, National Planning Commission Secretariat Central Bureau of Statistics, Kathmandu, Nepal. 2 Panchthar
3. GoV. 2013. National Sample Census of Agriculture, Nepal. Government of Nepal, National Planning Commission Secretariat Central Bureau of Statistics, Kathmandu, Nepal. 3 Illam
4. GoV. 2013. National Sample Census of Agriculture, Nepal. Government of Nepal, National Planning Commission Secretariat Central Bureau of Statistics, Kathmandu, Nepal. 4 Jhapa
5. GoV. 2013. National Sample Census of Agriculture, Nepal. Government of Nepal, National Planning Commission Secretariat Central Bureau of Statistics, Kathmandu, Nepal. 5 Morang
6. GoV. 2013. National Sample Census of Agriculture, Nepal. Government of Nepal, National Planning Commission Secretariat Central Bureau of Statistics, Kathmandu, Nepal. 6 Sunsari
7. GoV. 2013. National Sample Census of Agriculture, Nepal. Government of Nepal, National Planning Commission Secretariat Central Bureau of Statistics, Kathmandu, Nepal. 7 Dhankuta
8. GoV. 2013. National Sample Census of Agriculture, Nepal. Government of Nepal, National Planning Commission Secretariat Central Bureau of Statistics, Kathmandu, Nepal. 8 Terhathum
9. GoV. 2013. National Sample Census of Agriculture, Nepal. Government of Nepal, National Planning Commission Secretariat Central Bureau of Statistics, Kathmandu, Nepal. 9 Sankhuwasabha
10. GoV. 2013. National Sample Census of Agriculture, Nepal. Government of Nepal, National Planning Commission Secretariat Central Bureau of Statistics, Kathmandu, Nepal. 10 Bhojpur
11. GoV. 2013. National Sample Census of Agriculture, Nepal. Government of Nepal, National Planning Commission Secretariat Central Bureau of Statistics, Kathmandu, Nepal. 11 Solukhumbu
12. GoV. 2013. National Sample Census of Agriculture, Nepal. Government of Nepal, National Planning Commission Secretariat Central Bureau of Statistics, Kathmandu, Nepal. 12 Okhaldhunga
13. GoV. 2013. National Sample Census of Agriculture, Nepal. Government of Nepal, National Planning Commission Secretariat Central Bureau of Statistics, Kathmandu, Nepal. 13 Khotang
14. GoV. 2013. National Sample Census of Agriculture, Nepal. Government of Nepal, National Planning Commission Secretariat Central Bureau of Statistics, Kathmandu, Nepal. 14 Udayapur
15. GoV. 2013. National Sample Census of Agriculture, Nepal. Government of Nepal, National Planning Commission Secretariat Central Bureau of Statistics, Kathmandu, Nepal. 15 Saptari
16. GoV. 2013. National Sample Census of Agriculture, Nepal. Government of Nepal, National Planning Commission Secretariat Central Bureau of Statistics, Kathmandu, Nepal. 16 Siraha
17. GoV. 2013. National Sample Census of Agriculture, Nepal. Government of Nepal, National Planning Commission Secretariat Central Bureau of Statistics, Kathmandu, Nepal. 17 Dhanusa
18. GoV. 2013. National Sample Census of Agriculture, Nepal. Government of Nepal, National Planning Commission Secretariat Central Bureau of Statistics, Kathmandu, Nepal. 18 Mahottari
19. GoV. 2013. National Sample Census of Agriculture, Nepal. Government of Nepal, National Planning Commission Secretariat Central Bureau of Statistics, Kathmandu, Nepal. 19 Sarlahi
20. GoV. 2013. National Sample Census of Agriculture, Nepal. Government of Nepal, National Planning Commission Secretariat Central Bureau of Statistics, Kathmandu, Nepal. 20 Sindhuli
21. GoV. 2013. National Sample Census of Agriculture, Nepal. Government of Nepal, National Planning Commission Secretariat Central Bureau of Statistics, Kathmandu, Nepal. 21 Ramechhap
22. GoV. 2013. National Sample Census of Agriculture, Nepal. Government of Nepal, National Planning Commission Secretariat Central Bureau of Statistics, Kathmandu, Nepal. 22 Dolakha
23. GoV. 2013. National Sample Census of Agriculture, Nepal. Government of Nepal, National Planning Commission Secretariat Central Bureau of Statistics, Kathmandu, Nepal. 23 Sindhupalchowk
24. GoV. 2013. National Sample Census of Agriculture, Nepal. Government of Nepal, National Planning Commission Secretariat Central Bureau of Statistics, Kathmandu, Nepal. 24 Kavrepalanchowk
25. GoV. 2013. National Sample Census of Agriculture, Nepal. Government of Nepal, National Planning Commission Secretariat Central Bureau of Statistics, Kathmandu, Nepal. 25 Lalitpur
26. GoV. 2013. National Sample Census of Agriculture, Nepal. Government of Nepal, National Planning Commission Secretariat Central Bureau of Statistics, Kathmandu, Nepal. 26 Bhaktapur
27. GoV. 2013. National Sample Census of Agriculture, Nepal. Government of Nepal, National Planning Commission Secretariat Central Bureau of Statistics, Kathmandu, Nepal. 27 Kathmandu
28. GoV. 2013. National Sample Census of Agriculture, Nepal. Government of Nepal, National Planning Commission Secretariat Central Bureau of Statistics, Kathmandu, Nepal. 28 Nuwakot
29. GoV. 2013. National Sample Census of Agriculture, Nepal. Government of Nepal, National Planning Commission Secretariat Central Bureau of Statistics, Kathmandu, Nepal. 29 Rasuwa
30. GoV. 2013. National Sample Census of Agriculture, Nepal. Government of Nepal, National Planning Commission Secretariat Central Bureau of Statistics, Kathmandu, Nepal. 30 Dhadhing
31. GoV. 2013. National Sample Census of Agriculture, Nepal. Government of Nepal, National Planning Commission Secretariat Central Bureau of Statistics, Kathmandu, Nepal. 31 Makwanpur
32. GoV. 2013. National Sample Census of Agriculture, Nepal. Government of Nepal, National Planning Commission Secretariat Central Bureau of Statistics, Kathmandu, Nepal. 32 Rautahat
33. GoV. 2013. National Sample Census of Agriculture, Nepal. Government of Nepal, National Planning Commission Secretariat Central Bureau of Statistics, Kathmandu, Nepal. 33 Bara
34. GoV. 2013. National Sample Census of Agriculture, Nepal. Government of Nepal, National Planning Commission Secretariat Central Bureau of Statistics, Kathmandu, Nepal. 34 Parsa
35. GoV. 2013. National Sample Census of Agriculture, Nepal. Government of Nepal, National Planning Commission Secretariat Central Bureau of Statistics, Kathmandu, Nepal. 35 Chitwan
36. GoV. 2013. National Sample Census of Agriculture, Nepal. Government of Nepal, National Planning Commission Secretariat Central Bureau of Statistics, Kathmandu, Nepal. 36 Gorkha
37. GoV. 2013. National Sample Census of Agriculture, Nepal. Government of Nepal, National Planning Commission Secretariat Central Bureau of Statistics, Kathmandu, Nepal. 37 Lamjung
38. GoV. 2013. National Sample Census of Agriculture, Nepal. Government of Nepal, National Planning Commission Secretariat Central Bureau of Statistics, Kathmandu, Nepal. 38 Tanahu
39. GoV. 2013. National Sample Census of Agriculture, Nepal. Government of Nepal, National Planning Commission Secretariat Central Bureau of Statistics, Kathmandu, Nepal. 39 Syangja
40. GoV. 2013. National Sample Census of Agriculture, Nepal. Government of Nepal, National Planning Commission Secretariat Central Bureau of Statistics, Kathmandu, Nepal. 40 Kaski
41. GoV. 2013. National Sample Census of Agriculture, Nepal. Government of Nepal, National Planning Commission Secretariat Central Bureau of Statistics, Kathmandu, Nepal. 41 Manang
42. GoV. 2013. National Sample Census of Agriculture, Nepal. Government of Nepal, National Planning Commission Secretariat Central Bureau of Statistics, Kathmandu, Nepal. 42 Mustang
43. GoV. 2013. National Sample Census of Agriculture, Nepal. Government of Nepal, National Planning Commission Secretariat Central Bureau of Statistics, Kathmandu, Nepal. 43 Myagdi
44. GoV. 2013. National Sample Census of Agriculture, Nepal. Government of Nepal, National Planning Commission Secretariat Central Bureau of Statistics, Kathmandu, Nepal. 44 Parbat
45. GoV. 2013. National Sample Census of Agriculture, Nepal. Government of Nepal, National Planning Commission Secretariat Central Bureau of Statistics, Kathmandu, Nepal. 45 Baglung
46. GoV. 2013. National Sample Census of Agriculture, Nepal. Government of Nepal, National Planning Commission Secretariat Central Bureau of Statistics, Kathmandu, Nepal. 46 Gulmi
47. GoV. 2013. National Sample Census of Agriculture, Nepal. Government of Nepal, National Planning Commission Secretariat Central Bureau of Statistics, Kathmandu, Nepal. 47 Palpa
48. GoV. 2013. National Sample Census of Agriculture, Nepal. Government of Nepal, National Planning Commission Secretariat Central Bureau of Statistics, Kathmandu, Nepal. 48 Nawalparasi
49. GoV. 2013. National Sample Census of Agriculture, Nepal. Government of Nepal, National Planning Commission Secretariat Central Bureau of Statistics, Kathmandu, Nepal. 49 Rupendehi
50. GoV. 2013. National Sample Census of Agriculture, Nepal. Government of Nepal, National Planning Commission Secretariat Central Bureau of Statistics, Kathmandu, Nepal. 50 Kapilbastu
51. GoV. 2013. National Sample Census of Agriculture, Nepal. Government of Nepal, National Planning Commission Secretariat Central Bureau of Statistics, Kathmandu, Nepal. 51 Arghakhanchi
52. GoV. 2013. National Sample Census of Agriculture, Nepal. Government of Nepal, National Planning Commission Secretariat Central Bureau of Statistics, Kathmandu, Nepal. 52 Pyuthan
53. GoV. 2013. National Sample Census of Agriculture, Nepal. Government of Nepal, National Planning Commission Secretariat Central Bureau of Statistics, Kathmandu, Nepal. 53 Rolpa
54. GoV. 2013. National Sample Census of Agriculture, Nepal. Government of Nepal, National Planning Commission Secretariat Central Bureau of Statistics, Kathmandu, Nepal. 54 Rukum
55. GoV. 2013. National Sample Census of Agriculture, Nepal. Government of Nepal, National Planning Commission Secretariat Central Bureau of Statistics, Kathmandu, Nepal. 55 Salyan
56. GoV. 2013. National Sample Census of Agriculture, Nepal. Government of Nepal, National Planning Commission Secretariat Central Bureau of Statistics, Kathmandu, Nepal. 56 Dang
57. GoV. 2013. National Sample Census of Agriculture, Nepal. Government of Nepal, National Planning Commission Secretariat Central Bureau of Statistics, Kathmandu, Nepal. 57 Banke
58. GoV. 2013. National Sample Census of Agriculture, Nepal. Government of Nepal, National Planning Commission Secretariat Central Bureau of Statistics, Kathmandu, Nepal. 58 Bardiya
59. GoV. 2013. National Sample Census of Agriculture, Nepal. Government of Nepal, National Planning Commission Secretariat Central Bureau of Statistics, Kathmandu, Nepal. 59 Surkhet
60. GoV. 2013. National Sample Census of Agriculture, Nepal. Government of Nepal, National Planning Commission Secretariat Central Bureau of Statistics, Kathmandu, Nepal. 60 Dailekh
61. GoV. 2013. National Sample Census of Agriculture, Nepal. Government of Nepal, National Planning Commission Secretariat Central Bureau of Statistics, Kathmandu, Nepal. 61 Jajarkot
62. GoV. 2013. National Sample Census of Agriculture, Nepal. Government of Nepal, National Planning Commission Secretariat Central Bureau of Statistics, Kathmandu, Nepal. 62 Dolpa
63. GoV. 2013. National Sample Census of Agriculture, Nepal. Government of Nepal, National Planning Commission Secretariat Central Bureau of Statistics, Kathmandu, Nepal. 63 Jumla
64. GoV. 2013. National Sample Census of Agriculture, Nepal. Government of Nepal, National Planning Commission Secretariat Central Bureau of Statistics, Kathmandu, Nepal. 64 Kalikot
65. GoV. 2013. National Sample Census of Agriculture, Nepal. Government of Nepal, National Planning Commission Secretariat Central Bureau of Statistics, Kathmandu, Nepal. 65 Mugu
66. GoV. 2013. National Sample Census of Agriculture, Nepal. Government of Nepal, National Planning Commission Secretariat Central Bureau of Statistics, Kathmandu, Nepal. 66 Humla
67. GoV. 2013. National Sample Census of Agriculture, Nepal. Government of Nepal, National Planning Commission Secretariat Central Bureau of Statistics, Kathmandu, Nepal. 67 Bajura
68. GoV. 2013. National Sample Census of Agriculture, Nepal. Government of Nepal, National Planning Commission Secretariat Central Bureau of Statistics, Kathmandu, Nepal. 68 Bajhang
69. GoV. 2013. National Sample Census of Agriculture, Nepal. Government of Nepal, National Planning Commission Secretariat Central Bureau of Statistics, Kathmandu, Nepal. 69 Achham
70. GoV. 2013. National Sample Census of Agriculture, Nepal. Government of Nepal, National Planning Commission Secretariat Central Bureau of Statistics, Kathmandu, Nepal. 70 Doti
71. GoV. 2013. National Sample Census of Agriculture, Nepal. Government of Nepal, National Planning Commission Secretariat Central Bureau of Statistics, Kathmandu, Nepal. 71 Kailali
72. GoV. 2013. National Sample Census of Agriculture, Nepal. Government of Nepal, National Planning Commission Secretariat Central Bureau of Statistics, Kathmandu, Nepal. 72 Kanchanpur
73. GoV. 2013. National Sample Census of Agriculture, Nepal. Government of Nepal, National Planning Commission Secretariat Central Bureau of Statistics, Kathmandu, Nepal. 73 Dadeldhura
74. GoV. 2013. National Sample Census of Agriculture, Nepal. Government of Nepal, National Planning Commission Secretariat Central Bureau of Statistics, Kathmandu, Nepal. 74 Baitadi
75. GoV. 2013. National Sample Census of Agriculture, Nepal. Government of Nepal, National Planning Commission Secretariat Central Bureau of Statistics, Kathmandu, Nepal. 75 Darchula
76. MoAD, NTCDB, HELVETAS. 2014. Coffee database in Nepal [Internet]. Kathmandu: HELVETAS Nepal. Available: http://assets.helvetas.org/downloads/coffee_database_in_nepal__2014_.pdf
77. MoAD. 2014. Statistical information on Nepalese agriculture 2013/2014 [Internet]. Kathmandu, Nepal. Available: <http://www.moad.gov.np/en/content.php?id=332>
78. MOAD. 2012. Value chain development plan for organic coffee [Internet]. Kathmandu, Nepal. Available: http://pact.gov.np/docs/publication/Value Chain Development Plan for Organic Coffee.pdf
79. Ethirajan A. 2013. Nepal farmers brew success with coffee cultivation. In: BBC News, Nepal [Internet]. [cited 20 Dec 2014]. Available: http://www.bbc.com/news/business-21583681
80. NTCDB. Old Coffee Data. In: Coffee Statistics [Internet]. [cited 20 Dec 2014]. Available: http://www.teacoffee.gov.np/en/detail.php?section=coffee&nav_id=16&nav_name=coffee-statistics
81. Rauniyar I, Burke J. 2012. Nepalese farmers tap into global thirst for coffee. In: The Guardian [Internet]. [cited 20 Dec 2014]. Available: https://www.theguardian.com/world/2012/dec/18/nepal-farmers-coffee-industry-booming
