# Supplementary material for: Suitability Analysis and Projected Climate Change Impact on Banana and Coffee Production Zones in Nepal
Source: PLoS One. 2016 Sep 30;11(9):e0163916. doi: 10.1371/journal.pone.0163916 (PMC5045210; doi:10.1371/journal.pone.0163916)
Supplement: S1 Table — (DOC) [file pone.0163916.s003.doc]

**S1 Table. Global Environmental Stratification of Nepal**

| Sn | Broad biome | GEnZ | Abb (code*) | Lillesø (BPP) |
| --- | --- | --- | --- | --- |
| 1 | Alpine | Extremely cold and wet 1 | ECW1 (3) | Al (Al/Sa) |
| 2 | Alpine | Extremely cold and wet 2 | ECW2 (4) | Al/TH (Al/Sa) |
| 3 | Alpine | Cold and wet | CW (5) | Al (Al/Sa) |
| 4 | Alpine | Extremely cold and mesic | ECM (6) | Al/Sa (Al/Sa) |
| 5 | Alpine | Cold and mesic | CM (7) | Sa (Al/Sa) |
| 6 | Cool temperate | Cool temperate and dry | CTD (8) | Te (Te/St) |
| 7 | Cool temperate | Cool temperate and moist | CTM (10) | Te (Te/St) |
| 8 | Warm temperate | Warm temperate and mesic | WTM (11) | Te (Te/St) |
| 9 | Sub-tropical | Hot and mesic | HM (13) | Sub-tropical (Te/St) |
| 10 | Drylands | Hot and dry | HD (14) | Sub-tropical/Upper tropical (Tr) |
| 11 | Tropical | Extremely hot and moist | EHM (18) | Lower tropical (Tr) |

Abb: Abbreviation; Al: Alpine, TH: Trans-Himalayan, Sa: Sub-alpine, Te: Temperate, St: Sub-tropical and Tr: Tropical; Lillesø: based on Lillesø et al [1]; BPP: Based on Biodiversity profile project [2]

1. Lillesø J-PB, Shrestha TB, Dhakal LP, Nayaju RP, Shrestha R. The Map of Potential Vegetation of Nepal - a forestry/agro-ecological/biodiversity classifi cation system. Forest & Landscape Development and Environment Series 2-2005 and CFC-TIS Document Series No.110; 2005.

2. BPP. Biodiversity Profiles Project. Kathmandu, Nepal: Department of National Park and Wildlife Conservation, HMG/Nepal and Mountain Institute, Mount Everest Program; 1996.
