# Supplementary material for: Suitability Analysis and Projected Climate Change Impact on Banana and Coffee Production Zones in Nepal
Source: PLoS One. 2016 Sep 30;11(9):e0163916. doi: 10.1371/journal.pone.0163916 (PMC5045210; doi:10.1371/journal.pone.0163916)
Supplement: S2 Table — (DOC) [file pone.0163916.s004.doc]

**S2 Table. Bio-climatic and geophysical variables used in modelling process**

| **Sn** | **Abb** | **Name of variable** | **Source** |
| --- | --- | --- | --- |
| 1 | ***AI*** | ***Annual aridity index*** | [1] |
| 2 | bio_1 | Mean annual temperature | [2] |
| 3 | ***bio_2*** | ***Mean Diurnal temperature*** | [2] |
| 4 | ***bio_3*** | ***Isothermality*** | [2] |
| 5 | bio_4 | Temperature seasonality (standard deviation *100) | [2] |
| 6 | bio_5 | Max temperature of warmest month | [2] |
| 7 | bio_6 | Min temperature of coldest month | [2] |
| 8 | bio_7 | Temperature annual range (Bio5–Bio6) | [2] |
| 9 | bio_8 | Mean temperature of the wettest quarter | [2] |
| 10 | bio_9 | Mean temperature of driest quarter | [2] |
| 11 | bio_10 | Mean temperature of warmest quarter | [2] |
| 12 | ***LC*** | ***Land cover of Nepal**** | [3] |
| 12 | bio_11 | Mean temperature of coldest quarter | [2] |
| 13 | bio_12 | Total (annual) precipitation | [2] |
| 14 | bio_13 | Precipitation of wettest month | [2] |
| 15 | ***bio_14*** | ***Precipitation of Driest month*** | [2] |
| 16 | ***bio_15*** | ***Precipitation seasonality*** | [2] |
| 17 | bio_16 | Precipitation of wettest quarter | [2] |
| 18 | bio_17 | Precipitation of driest quarter | [2] |
| 19 | ***bio_18*** | ***Precipitation of Warmest Quarter*** | [2] |
| 20 | ***bio_19*** | ***Precipitation of Coldest Quarter*** | [2] |
| 21 | pet_spr | Potential evapo-transpiration for spring months | [1] |
| 22 | ***pet_sum*** | ***Potential evapo-transpiration for summer months*** | [1] |
| 23 | pet_aut | Potential evapo-transpiration for autumn months | [1] |
| 24 | pet_win | Potential evapo-transpiration for winter months | [1] |
| 25 | t_dd | Temperature degree day | [2] |
| 26 | ***GEnS*** | ***Global Environmental Stratification*** | [4] |
| 27 | ***ASP*** | ***Aspect*** | [5] |
| 28 | ***Slo*** | ***Slope*** | [5] |

Bold-italic variables are used in final model selected based on VIF analysis. *Land cover is used as dummy variable, details category of LC of Nepal is provided in Table A.2

1. Zomer RJ, Trabucco A, Bossio D a., Verchot L V. Climate change mitigation: A spatial analysis of global land suitability for clean development mechanism afforestation and reforestation. Agric Ecosyst Environ. 2008;126: 67–80. doi:10.1016/j.agee.2008.01.014

2. Hijmans RJ, Cameron SE, Parra JL, Jones PG, Jarvis A. Very high resolution interpolated climate surface for global land areas. Int J Climatol. 2005;25: 1965–1978. doi:10.1002/joc.1276

3. Uddin K, Shrestha HL, Murthy MSR, Bajracharya B, Shrestha B, Gilani H, et al. Development of 2010 national land cover database for the Nepal. J Environ Manage. Elsevier Ltd; 2015;148: 82–90. doi:10.1016/j.jenvman.2014.07.047

4. Metzger MJ, Bunce RGH, Jongman RHG, Sayre R, Trabucco A, Zomer R. A high-resolution bioclimate map of the world: a unifying framework for global biodiversity research and monitoring. Sykes M, editor. Glob Ecol Biogeogr. 2013;22: 630–638. doi:10.1111/geb.12022

5. Jarvis A, Reuter HI, Nelson A, Guevara E. Hole-filled SRTM for the globe Version 4 [Internet]. 2008 [cited 15 Jan 2015]. Available: http://www.cgiar-csi.org/data/srtm-90m-digital-elevation-database-v4-1
