# Supplementary material for: Suitability Analysis and Projected Climate Change Impact on Banana and Coffee Production Zones in Nepal
Source: PLoS One. 2016 Sep 30;11(9):e0163916. doi: 10.1371/journal.pone.0163916 (PMC5045210; doi:10.1371/journal.pone.0163916)
Supplement: S3 Table — (DOC) [file pone.0163916.s005.doc]

**S3 Table. Variance Inflation Factor (VIF) in different test runs for selection of explanatory variables and correlation between predictor variables.** Each subsequent test run (M1, M2,…M17) excluded the variable with the highest VIF (highlighted in yellow); while remaining variable (with bold letters) were selected.

**Banana**

| **Variables** | **M1** | **M2** | **M3** | **M4** | **M5** | **M6** | **M7** | **M8** | **M9** | **M10** | **M11** | **M12** | **M13** | **M14** | **M15** | **M16** | **M17** | **Final** |
| --- | --- | --- | --- | --- | --- | --- | --- | --- | --- | --- | --- | --- | --- | --- | --- | --- | --- | --- |
| AI | 53.19 | 53.19 | 52.32 | 47.61 | 47.61 | 47.19 | 45.59 | 45.39 | 38.32 | 38.28 | 37.68 | 22.31 | 22.3 | 19.14 | 19.1 | 9.16 | 9.13 | **8.49** |
| bio_2 | 180.69 | 180.69 | 178.43 | 173.52 | 173.49 | 173.02 | 166.91 | 166.21 | 164.19 | 163.61 | 23.36 | 21.83 | 21.77 | 20.14 | 20.12 | 16.33 | 15.86 | **2.4** |
| bio_3 | 36.73 | 36.73 | 36.73 | 36.02 | 35.41 | 35.12 | 35.08 | 34.7 | 34.62 | 34.53 | 13.45 | 13.45 | 13.44 | 13.41 | 13.38 | 11.16 | 9.87 | **2.82** |
| bio_14 | 4.67 | 4.67 | 4.61 | 4.6 | 4.56 | 4.47 | 4.43 | 3.79 | 3.79 | 3.69 | 3.4 | 3.38 | 3.35 | 3.3 | 2.45 | 2.43 | 2.26 | **2.25** |
| bio_15 | 29.11 | 29.11 | 27.83 | 26.59 | 23.52 | 22.34 | 22.25 | 18.44 | 14.94 | 14.92 | 14.77 | 11.82 | 11.7 | 11.67 | 8.13 | 7.95 | 7.69 | **7.32** |
| bio_17 | 54.81 | 54.81 | 54.8 | 53.61 | 52.44 | 52.3 | 51.67 | 51.67 | 51.55 | 50.75 | 49.41 | 48.46 | 38.98 | 38.41 | 9.91 | 9.82 | 5.72 | 5.68 |
| bio_18 | 18.95 | 18.95 | 18.86 | 18.78 | 18.77 | 18.65 | 18.62 | 18.61 | 18.51 | 18.28 | 16.31 | 15.67 | 15.49 | 15.49 | 15.49 | 12.01 | 10.89 | **10.18** |
| pet_sum | 431.79 | 431.79 | 427.42 | 378.55 | 369.81 | 160.82 | 147.59 | 141.53 | 133.17 | 44.93 | 44.86 | 38.78 | 37.86 | 34.81 | 33.83 | 30.66 | 5 | **4.69** |
| bio_4 | 291.64 | 291.64 | 91.22 | 82.69 | 49.88 | 48.53 | 37.57 | 36.51 | 35.48 | 30.95 | 22.2 | 21.23 | 20.17 | 20.09 | 19.69 | 17.65 | 16.13 |  |
| pet_win | 792.17 | 792.17 | 790.54 | 783.33 | 666.09 | 205.51 | 129.42 | 119.07 | 104.94 | 77.28 | 77.19 | 70.78 | 63.26 | 39.08 | 35.91 | 34.66 |  |  |
| bio_13 | 185.19 | 185.19 | 185.09 | 184.98 | 184.98 | 184.9 | 174.01 | 143.22 | 135.17 | 122.61 | 117.78 | 50.84 | 50 | 43.77 | 42.74 |  |  |  |
| bio_19 | 76.22 | 76.22 | 75.98 | 75.67 | 68.71 | 68.45 | 64.6 | 63.92 | 63.82 | 63.73 | 63.73 | 61.93 | 46.56 | 44.84 |  |  |  |  |
| bio_9 | 133.8 | 133.8 | 133.19 | 133.08 | 128.09 | 127.56 | 118.83 | 116.86 | 102.7 | 101.12 | 98.13 | 96.57 | 64.04 |  |  |  |  |  |
| t_dd | 221.64 | 221.64 | 205.16 | 202.67 | 198.96 | 198.16 | 176.36 | 169.81 | 113.38 | 111.73 | 111.62 | 107.88 |  |  |  |  |  |  |
| bio_12 | 671.56 | 671.56 | 639.55 | 633.85 | 610.25 | 601.48 | 601.48 | 222.37 | 192.81 | 182.62 | 179.29 |  |  |  |  |  |  |  |
| bio_7 | Inf | 290.22 | 289.59 | 286.62 | 258.42 | 254.7 | 252.65 | 252.63 | 252.49 | 242.14 |  |  |  |  |  |  |  |  |
| pet_spr | 470.71 | 470.71 | 466.56 | 453.12 | 415.04 | 412.91 | 370.27 | 324.65 | 322.92 |  |  |  |  |  |  |  |  |  |
| bio_8 | 1586.52 | 1586.52 | 1576.64 | 1480.18 | 1174.74 | 1103.7 | 352.82 | 351.89 |  |  |  |  |  |  |  |  |  |  |
| bio_16 | 691.77 | 691.77 | 669.84 | 669.82 | 639.71 | 628.47 | 625.43 |  |  |  |  |  |  |  |  |  |  |  |
| bio_6 | Inf | 5464.46 | 4626.16 | 4259.2 | 1382.95 | 1342.04 |  |  |  |  |  |  |  |  |  |  |  |  |
| pet_aut | 1475.28 | 1475.28 | 1466.98 | 1455.81 | 1455.62 |  |  |  |  |  |  |  |  |  |  |  |  |  |
| bio_10 | 13750.52 | 13750.52 | 10220.91 | 5567.09 |  |  |  |  |  |  |  |  |  |  |  |  |  |  |
| bio_1 | 26842.4 | 26842.4 | 10837.61 |  |  |  |  |  |  |  |  |  |  |  |  |  |  |  |
| bio_11 | 50370.13 | 50370.13 |  |  |  |  |  |  |  |  |  |  |  |  |  |  |  |  |
| bio_5 | Inf |  |  |  |  |  |  |  |  |  |  |  |  |  |  |  |  |  |

Inf – variable with very high collinearity; bio_14 and bio_17 are highly correlated therefore we keep bio_14 (lower VIF) in the final model

Coffee

| **Variables** | **M1** | **M2** | **M3** | **M4** | **M5** | **M6** | **M7** | **M8** | **M9** | **M10** | **M11** | **M12** | **M13** | **M14** | **M15** | **M16** | **M17** | **Final** |
| --- | --- | --- | --- | --- | --- | --- | --- | --- | --- | --- | --- | --- | --- | --- | --- | --- | --- | --- |
| AI | 172.19 | 172.19 | 172.17 | 171.98 | 170.15 | 167.05 | 167 | 124.41 | 123.18 | 113.94 | 74.02 | 73.71 | 30.26 | 30.18 | 29.93 | 3.89 | 3.86 | **3.86** |
| bio_14 | 5.26 | 5.26 | 5.18 | 4.91 | 4.67 | 4.64 | 4.3 | 3.58 | 3.58 | 3.48 | 3.3 | 3.27 | 3.27 | 3.11 | 1.82 | 1.81 | 1.75 | **1.73** |
| bio_15 | 23.27 | 23.27 | 22.47 | 22.38 | 22.07 | 21.48 | 20.95 | 12.48 | 11.94 | 7.05 | 6.99 | 6.87 | 6.86 | 6.71 | 6.53 | 6.5 | 6.49 | **6.38** |
| bio_19 | 36.75 | 36.75 | 36.52 | 36.48 | 35.82 | 35.81 | 35.78 | 35.76 | 35.7 | 34.24 | 33.73 | 33.54 | 31.95 | 30.81 | 4.63 | 4.61 | 4.22 | **3.98** |
| bio_2 | 89.89 | 89.89 | 87.97 | 85.95 | 85.94 | 84.6 | 84.3 | 82.02 | 75.47 | 75.04 | 72.68 | 21.48 | 14.24 | 12.43 | 11.99 | 11.93 | 11.17 | **2.46** |
| bio_3 | 27.14 | 27.14 | 27.11 | 27.1 | 26.98 | 26.92 | 26.86 | 26.82 | 26.59 | 26.44 | 26.44 | 10.66 | 9.14 | 8.84 | 8.8 | 7.87 | 7.59 | **2.36** |
| pet_sum | 439.87 | 439.87 | 430.73 | 420.43 | 419.4 | 415.47 | 178.73 | 156.93 | 61.5 | 47.08 | 41.63 | 41.14 | 39.13 | 27.57 | 27.32 | 27.05 | 2.87 | **2.66** |
| bio_4 | 201.3 | 201.3 | 110.81 | 80.64 | 80.45 | 53.64 | 53.6 | 53.35 | 51.26 | 32.99 | 31.95 | 26.19 | 23 | 19.74 | 18.84 | 18.83 | 18.82 |  |
| bio_9 | 367.68 | 367.68 | 362.82 | 362.66 | 362.39 | 311.86 | 309.77 | 298.14 | 293.66 | 77.39 | 62.74 | 62.51 | 51.23 | 29.95 | 29.94 | 29.11 |  |  |
| bio_18 | 42.65 | 42.65 | 41.64 | 40.93 | 39.94 | 38.23 | 38.16 | 36.38 | 36.15 | 36.15 | 36.1 | 34.56 | 33.01 | 32.19 | 32.15 |  |  |  |
| bio_17 | 42.77 | 42.77 | 42.56 | 41.81 | 41.2 | 41.16 | 41.16 | 41.14 | 41.12 | 39.79 | 39.62 | 39.5 | 37.79 | 37.79 |  |  |  |  |
| pet_win | 1169.84 | 1169.84 | 1142.89 | 1066.7 | 714.92 | 625.65 | 163.93 | 153.42 | 87.63 | 85.42 | 74.47 | 73.44 | 71.16 |  |  |  |  |  |
| bio_13 | 204.75 | 204.75 | 204.74 | 204.61 | 196.21 | 173.89 | 171.89 | 171.35 | 170.42 | 167.9 | 83.66 | 80.87 |  |  |  |  |  |  |
| bio_7 | Inf | 424.91 | 372.49 | 349.01 | 305.53 | 208.53 | 203.86 | 203.46 | 180.81 | 152.01 | 151.88 |  |  |  |  |  |  |  |
| bio_16 | 1002.67 | 1002.67 | 953.46 | 952.95 | 904.32 | 901.54 | 901.15 | 298.86 | 294.8 | 280.34 |  |  |  |  |  |  |  |  |
| bio_8 | 3007.36 | 3007.36 | 2967.89 | 2540.1 | 1919.08 | 424.64 | 422.44 | 353.76 | 351.36 |  |  |  |  |  |  |  |  |  |
| pet_spr | 766.14 | 766.14 | 726.31 | 720.53 | 515.25 | 465.07 | 461.13 | 383.82 |  |  |  |  |  |  |  |  |  |  |
| bio_12 | 1034.32 | 1034.32 | 963.95 | 962.65 | 960.7 | 960.6 | 954.62 |  |  |  |  |  |  |  |  |  |  |  |
| pet_aut | 1220.98 | 1220.98 | 1195.51 | 1191.45 | 1189.52 | 1189.31 |  |  |  |  |  |  |  |  |  |  |  |  |
| bio_5 | Inf | 9179.63 | 7523.02 | 6606.73 | 2339.17 |  |  |  |  |  |  |  |  |  |  |  |  |  |
| bio_1 | 20003.06 | 20003.06 | 12402.04 | 7981.53 |  |  |  |  |  |  |  |  |  |  |  |  |  |  |
| bio_10 | 15807.46 | 15807.46 | 13916.34 |  |  |  |  |  |  |  |  |  |  |  |  |  |  |  |
| bio_11 | 32026.23 | 32026.23 |  |  |  |  |  |  |  |  |  |  |  |  |  |  |  |  |
| bio_5 | Inf |  |  |  |  |  |  |  |  |  |  |  |  |  |  |  |  |  |
