# Supplementary material for: Suitability Analysis and Projected Climate Change Impact on Banana and Coffee Production Zones in Nepal
Source: PLoS One. 2016 Sep 30;11(9):e0163916. doi: 10.1371/journal.pone.0163916 (PMC5045210; doi:10.1371/journal.pone.0163916)
Supplement: S4 Table — (DOC) [file pone.0163916.s006.doc]

**S4 Table. Land cover of Nepal** [1]

| **Value** | **Class name** | **Area** | |
| --- | --- | --- | --- |
|  |  | **Km^2^** | **Percent** |
| 1 | ***Needle-leaved closed forest*** | 13910.97 | 9.45 |
| 2 | ***Needle-leaved open forest*** | 8245.99 | 5.60 |
| 3 | ***Broadleaved closed forest*** | 21048.89 | 14.30 |
| 4 | ***Broadleaved open forest*** | 14043.52 | 9.54 |
| 5 | ***Shrubland*** | 4869.66 | 3.31 |
| 6 | ***Grassland*** | 11652.30 | 7.92 |
| 7 | ***Agriculture*** | 43713.11 | 29.70 |
| 8 | ***Barren area*** | 15733.58 | 10.69 |
| 9 | Built-up area | 463.68 | 0.32 |
| 10 | River | 832.81 | 0.57 |
| 11 | Lake | 45.46 | 0.03 |
| 12 | ***Snow/glacier*** | 12621.04 | 8.58 |
|  |  | **147181.00** | **100.00** |

Bold-italic classes are used in final model as dummy variable.

Source: Uddin et al. [1]

<http://apps.geoportal.icimod.org/ArcGIS/rest/services/Nepal/Landcover2010/MapServer/0>

**References**

1. Uddin K, Shrestha HL, Murthy MSR, Bajracharya B, Shrestha B, Gilani H, et al. Development of 2010 national land cover database for the Nepal. J Environ Manage. Elsevier Ltd; 2015;148: 82–90. doi:10.1016/j.jenvman.2014.07.047
