# Supplementary material for: Suitability Analysis and Projected Climate Change Impact on Banana and Coffee Production Zones in Nepal
Source: PLoS One. 2016 Sep 30;11(9):e0163916. doi: 10.1371/journal.pone.0163916 (PMC5045210; doi:10.1371/journal.pone.0163916)
Supplement: S5 Table — (DOC) [file pone.0163916.s007.doc]

**S5 Table. Species distribution algorithms included in ensemble modeling in the BiodiversityR package and used in the present analysis**

| **Sn** | **Algorithm and abbreviation** | **R-package** | **Citation** |
| --- | --- | --- | --- |
| 1 | Artificial neural networks (NNET) | nnet package | (Venables & Ripley 2002) |
| 2 | BIOCLIM | dismo package | (Hijmans et al. 2013) |
| 3 | DOMAIN | dismo package | (Hijmans et al. 2013); (Carpenter et al. 2003) |
| 4 | Flexible discriminant analysis (FDA) | mda package | (Hastie et al. 2011); (Hastie et al. 1994) |
| 5  6 | Generalized additive models (a) GAM  (b) GAMstep | gam package | (Hastie & Tibshirani 1990); (Hastie 2013) |
| 7  8 | (c) MGCV  (d) MGCVfix | mgcv package | (Wood 2006) |
| 9 | Generalized boosted regression models (GBM) | gbm package | (Ridgeway & Others 2013); (Ridgeway 1999) |
| 10 | Generalized linear models (GLM) | stats package | (R Development Core Team 2012) |
| 11 | Mahalanobis model (Mahal) | dismo package | (Hijmans et al. 2013) |
| 12 | Maximum entropy (Maxent) | dismo package | (Hijmans et al. 2013); (Phillips et al. 2006) |
| 13 | Multivariate adaptive regression splines (Earth) | earth package | (Milborrow & Wrapper. 2013); (Leathwick et al. 2005) |
| 14 | Random forests (RF) | randomForest package | (Breiman 2001); (Liaw & Wiener 2002) |
| 15 | Recursive partitioning and regression trees (RPart) | rpart package | (Therneau et al. 2013); (Breiman et al. 1984) |
| 16 | Stepwise boosted regression tree models (stepGBM) | dismo package | (Hijmans et al. 2013); (Elith et al. 2009) |
| 17 | Stepwise generalized linear models (stepGLM) | MASS package | (Venables & Ripley 2002) |
| 18 | Support vector machines (a) SVM | kernlab package | (Karatzoglou et al. 2004) |
| 19 | (b) SVME | e1071 package | (Meyer et al. 2012) |

*Algorithm not used for model calibration in present work

**Bibliography for R-packages used in BiodiversityR**

Breiman, L. 2001. Random Forests. Machine Learning **45**:5–32.

Breiman, L., J. H. Friedman, R. A. Olshen, and C. J. Stone. 1984. Classification and Regression Trees. .Wadsworth.

Carpenter, G., A. N. Gillison, and J. Winter. 2003. Domain: a flexible modelling procedure for mapping potential distributions of plants and animals. Biodiversity Conservation **2**:667–680.

Elith, J., J. R. Leathwick, and T. Hastie. 2009. A working guide to boosted regression trees. Journal of Animal Ecology **77**:802–81.

Hastie, T. 2013. gam: Generalized Additive Models. Available from http://cran.r-project.org/package=gam.

Hastie, T., and R. Tibshirani. 1990. Generalized Additive Models. London: Chapman and Hall.

Hastie, T., R. Tibshirani, and A. Buja. 1994. Flexible Disriminant Analysis by Optimal Scoring. Journal of the American Statistical Association:1255–1270.

Hastie, T., R. Tibshirani, F. Leisch, K. Hornik, and B. D. Ripley. 2011. mda: Mixture and flexible discriminant analysis. S original by Trevor Hastie and Robert Tibshirani. R port by Friedrich Leisch and Kurt Hornik and Brian D. Repley.

Hijmans, R. J., S. Phillips, J. Leathwick, and J. Elith. 2013. dismo: Species distribution modeling. Available from http://cran.r-project.org/package=dismo.

Karatzoglou, A., A. Smola, K. Hornik, and A. Zeileis. 2004. kernlab - An S4 Package for Kernel Methods in R. Journal of Statistical Software **11**:1–20. Available from http://www.jstatsoft.org/v11/i09/.

Leathwick, J. R., D. Rowe, J. Richardson, J. Elith, and T. Hastie. 2005. Using multivariate adaptive regression splines to predict the distributions of New Zealand’s freshwater diadromous fish. Freshwater Biology **50**:2034–2052.

Liaw, A., and M. Wiener. 2002. Classification and Regression by randomForest. R News **2**:18–22.

Meyer, D., E. Dimitriadou, K. Hornik, A. Weingessel, and F. Leisch. 2012. e1071: Misc Functions of the Department of Statistics (e1071), TU Wien. Available from http://CRAN.R-project.org/package=e1071.

Milborrow, S., and D. from mda:mars by T. H. and R. T. U. A. M. F. utilities with T. L. leaps Wrapper. 2013. earth: Multivariate Adaptive Regression Spline Models. Available from http://cran.r-project.org/package=e.

Phillips, S. J., R. P. Anderson, and R. E. Schapire. 2006. Maximum entropy modeling of species geographic distributions. Ecological Modelling **190**:231–259. Available from http://linkinghub.elsevier.com/retrieve/pii/S030438000500267X (accessed March 8, 2012).

R Development Core Team. 2012. R: A language and environment for statistical computing. R Foundation for Statistical Computing, Vienna, Austria. Available from URL http://www.R-project.org/.

Ridgeway, G. 1999. The state of boosting. Computing Science and Statistics **31**:172–181.

Ridgeway, G., and with contributions from Others. 2013. gbm: Generalized Boosted Regression Models. Available from http://cran.r-project.org/package=gbm.

Therneau, T., B. Atkinson, and B. Ripley. 2013. rpart: Recursive Partitioning. Available from http://cran.r-project.org/package=rpart.

Venables, W. N., and B. D. Ripley. 2002. Modern Applied Statistics with SFourth Edi. Springer, New York.

Wood, S. N. 2006. Generalized Additive Models: An Introduction with R. Chapman and Hall/CRC.
