# Supplementary material for: Suitability Analysis and Projected Climate Change Impact on Banana and Coffee Production Zones in Nepal
Source: PLoS One. 2016 Sep 30;11(9):e0163916. doi: 10.1371/journal.pone.0163916 (PMC5045210; doi:10.1371/journal.pone.0163916)
Supplement: S6 Table — (DOC) [file pone.0163916.s008.doc]

**S6 Table. cAUC for sub-models, null model and ensemble model in the different model calibration**

**For banana**

| Models\Run | 1 | 2 | 3 | 4 | 5 | 6 | 7 | 8 | 9 | 10 | 11 | 12 | 13 | 14 | 15 | 16 | 17 | 18 | 19 | 20* |
| --- | --- | --- | --- | --- | --- | --- | --- | --- | --- | --- | --- | --- | --- | --- | --- | --- | --- | --- | --- | --- |
| NULL | 0.49 | 0.50 | 0.50 | 0.49 | 0.51 | 0.50 | 0.51 | 0.48 | 0.50 | 0.48 | 0.51 | 0.50 | 0.49 | 0.51 | 0.49 | 0.50 | 0.50 | 0.50 | 0.51 | 0.49 |
| Maxent | 0.56 | 0.55 | 0.55 | 0.59 | 0.57 | 0.57 | 0.63 | 0.64 | 0.58 | 0.67 | 0.68 | 0.72 | 0.69 | 0.70 | 0.69 | 0.70 | 0.64 | 0.69 | 0.73 | 0.72 |
| GBM | 0.57 | 0.53 | 0.60 | 0.57 | 0.57 | 0.58 | 0.61 | 0.60 | 0.65 | 0.60 | 0.65 | 0.67 | 0.68 | 0.70 | 0.66 | 0.72 | 0.64 | 0.70 | 0.71 | 0.71 |
| stepGBM | 0.50 | 0.54 | 0.58 | 0.49 | 0.51 | 0.58 | 0.58 | 0.54 | 0.62 | 0.58 | 0.61 | 0.66 | 0.64 | 0.68 | 0.65 | 0.66 | 0.63 | 0.72 | 0.67 | 0.67 |
| RF | 0.49 | 0.52 | 0.53 | 0.50 | 0.57 | 0.61 | 0.56 | 0.54 | 0.63 | 0.56 | 0.62 | 0.63 | 0.64 | 0.64 | 0.61 | 0.68 | 0.62 | 0.67 | 0.64 | 0.69 |
| GLM | 0.51 | 0.58 | 0.53 | 0.57 | 0.48 | 0.62 | 0.61 | 0.66 | 0.59 | 0.63 | 0.69 | 0.69 | 0.68 | 0.69 | 0.72 | 0.72 | 0.66 | 0.72 | 0.74 | 0.76 |
| stepGLM | 0.90 | 0.90 | 0.90 | 0.89 | 0.88 | 0.90 | 0.87 | 0.89 | 0.89 | 0.89 | 0.87 | 0.90 | 0.89 | 0.68 | 0.66 | 0.70 | 0.66 | 0.71 | 0.88 | 0.88 |
| GAM | 0.48 | 0.61 | 0.60 | 0.58 | 0.59 | 0.65 | 0.62 | 0.65 | 0.62 | 0.63 | 0.71 | 0.74 | 0.71 | 0.70 | 0.65 | 0.72 | 0.70 | 0.66 | 0.80 | 0.78 |
| stepGAM | 0.49 | 0.60 | 0.60 | 0.62 | 0.58 | 0.65 | 0.61 | 0.63 | 0.60 | 0.64 | 0.71 | 0.75 | 0.69 | 0.69 | 0.63 | 0.73 | 0.70 | 0.67 | 0.79 | 0.78 |
| MGCV | 0.51 | 0.57 | 0.55 | 0.60 | 0.51 | 0.62 | 0.58 | 0.67 | 0.59 | 0.63 | 0.73 | 0.71 | 0.72 | 0.69 | 0.69 | 0.71 | 0.69 | 0.71 | 0.74 | 0.77 |
| EARTH | 0.53 | 0.55 | 0.62 | 0.56 | 0.51 | 0.66 | 0.64 | 0.60 | 0.60 | 0.64 | 0.58 | 0.64 | 0.63 | 0.68 | 0.59 | 0.67 | 0.62 | 0.64 | 0.75 | 0.71 |
| RPART | 0.45 | 0.50 | 0.45 | 0.53 | 0.58 | 0.56 | 0.50 | 0.59 | 0.63 | 0.57 | 0.56 | 0.62 | 0.62 | 0.66 | 0.62 | 0.65 | 0.62 | 0.65 | 0.71 | 0.69 |
| NNET | 0.58 | 0.49 | 0.52 | 0.60 | 0.54 | 0.56 | 0.58 | 0.50 | 0.47 | 0.50 | 0.57 | 0.52 | 0.62 | 0.63 | 0.58 | 0.50 | 0.63 | 0.50 | 0.53 | 0.55 |
| FDA | 0.58 | 0.56 | 0.61 | 0.53 | 0.56 | 0.62 | 0.63 | 0.61 | 0.62 | 0.62 | 0.70 | 0.62 | 0.70 | 0.71 | 0.64 | 0.65 | 0.59 | 0.66 | 0.77 | 0.69 |
| SVM | 0.55 | 0.49 | 0.49 | 0.61 | 0.50 | 0.62 | 0.61 | 0.60 | 0.61 | 0.57 | 0.69 | 0.57 | 0.66 | 0.70 | 0.63 | 0.68 | 0.71 | 0.65 | 0.68 | 0.68 |
| SVME | 0.57 | 0.56 | 0.49 | 0.63 | 0.54 | 0.61 | 0.57 | 0.59 | 0.59 | 0.53 | 0.69 | 0.58 | 0.65 | 0.63 | 0.65 | 0.68 | 0.70 | 0.64 | 0.65 | 0.74 |
| BIOCLIM | 0.59 | 0.49 | 0.52 | 0.55 | 0.56 | 0.49 | 0.70 | 0.70 | 0.59 | 0.67 | 0.53 | 0.60 | 0.65 | 0.67 | 0.47 | 0.56 | 0.59 | 0.61 | 0.62 | 0.57 |
| DOMAIN | 0.63 | 0.56 | 0.52 | 0.61 | 0.62 | 0.51 | 0.66 | 0.68 | 0.65 | 0.67 | 0.44 | 0.62 | 0.63 | 0.64 | 0.51 | 0.52 | 0.51 | 0.57 | 0.59 | 0.62 |
| Mahal | 0.51 | 0.62 | 0.57 | 0.52 | 0.55 | 0.47 | 0.64 | 0.65 | 0.65 | 0.65 | 0.59 | 0.61 | 0.63 | 0.61 | 0.55 | 0.55 | 0.56 | 0.59 | 0.61 | 0.56 |
| Ensemble | 0.55 | 0.56 | 0.57 | 0.58 | 0.59 | 0.62 | 0.63 | 0.64 | 0.64 | 0.65 | 0.68 | 0.70 | 0.70 | 0.71 | 0.72 | 0.72 | 0.72 | 0.72 | 0.75 | 0.76 |

* Value of this calibration is used for further analysis

**For coffee**

| Models\Run | 1 | 2 | 3 | 4 | 5 | 6 | 7 | 8 | 9 | 10 | 11 | 12 | 13 | 14 | 15 | 16 | 17 | 18 | 19 | 20* |
| --- | --- | --- | --- | --- | --- | --- | --- | --- | --- | --- | --- | --- | --- | --- | --- | --- | --- | --- | --- | --- |
| NULL | 0.51 | 0.50 | 0.51 | 0.49 | 0.50 | 0.50 | 0.50 | 0.50 | 0.51 | 0.50 | 0.51 | 0.50 | 0.51 | 0.52 | 0.49 | 0.50 | 0.50 | 0.49 | 0.49 | 0.51 |
| Maxent | 0.54 | 0.56 | 0.67 | 0.57 | 0.60 | 0.63 | 0.59 | 0.61 | 0.60 | 0.67 | 0.70 | 0.66 | 0.51 | 0.57 | 0.59 | 0.63 | 0.70 | 0.61 | 0.67 | 0.80 |
| GBM | 0.63 | 0.62 | 0.60 | 0.65 | 0.63 | 0.64 | 0.66 | 0.66 | 0.61 | 0.61 | 0.64 | 0.66 | 0.67 | 0.73 | 0.70 | 0.74 | 0.76 | 0.70 | 0.73 | 0.80 |
| stepGBM | 0.63 | 0.65 | 0.61 | 0.64 | 0.65 | 0.66 | 0.69 | 0.66 | 0.63 | 0.64 | 0.68 | 0.69 | 0.67 | 0.73 | 0.69 | 0.78 | 0.77 | 0.71 | 0.74 | 0.78 |
| RF | 0.59 | 0.62 | 0.63 | 0.64 | 0.64 | 0.60 | 0.62 | 0.62 | 0.62 | 0.66 | 0.63 | 0.67 | 0.62 | 0.69 | 0.69 | 0.79 | 0.68 | 0.65 | 0.75 | 0.70 |
| GLM | 0.57 | 0.56 | 0.60 | 0.59 | 0.59 | 0.65 | 0.68 | 0.56 | 0.66 | 0.64 | 0.65 | 0.60 | 0.68 | 0.60 | 0.62 | 0.58 | 0.63 | 0.63 | 0.74 | 0.75 |
| stepGLM | 0.62 | 0.58 | 0.59 | 0.61 | 0.58 | 0.61 | 0.60 | 0.53 | 0.64 | 0.61 | 0.66 | 0.57 | 0.62 | 0.65 | 0.62 | 0.60 | 0.65 | 0.69 | 0.74 | 0.79 |
| GAM | 0.57 | 0.48 | 0.57 | 0.57 | 0.63 | 0.59 | 0.49 | 0.53 | 0.61 | 0.63 | 0.59 | 0.59 | 0.57 | 0.57 | 0.66 | 0.57 | 0.63 | 0.58 | 0.70 | 0.70 |
| stepGAM | 0.54 | 0.48 | 0.60 | 0.53 | 0.62 | 0.60 | 0.48 | 0.52 | 0.57 | 0.62 | 0.61 | 0.57 | 0.57 | 0.60 | 0.68 | 0.58 | 0.61 | 0.58 | 0.69 | 0.73 |
| MGCV | 0.57 | 0.55 | 0.53 | 0.61 | 0.57 | 0.63 | 0.67 | 0.57 | 0.68 | 0.64 | 0.65 | 0.65 | 0.68 | 0.56 | 0.61 | 0.57 | 0.65 | 0.63 | 0.72 | 0.76 |
| EARTH | 0.61 | 0.58 | 0.63 | 0.54 | 0.54 | 0.61 | 0.64 | 0.65 | 0.58 | 0.63 | 0.59 | 0.70 | 0.59 | 0.57 | 0.63 | 0.60 | 0.72 | 0.69 | 0.77 | 0.76 |
| RPART | 0.58 | 0.47 | 0.64 | 0.62 | 0.57 | 0.50 | 0.53 | 0.52 | 0.53 | 0.63 | 0.65 | 0.57 | 0.58 | 0.54 | 0.64 | 0.65 | 0.74 | 0.69 | 0.52 | 0.58 |
| NNET | 0.55 | 0.42 | 0.47 | 0.62 | 0.44 | 0.50 | 0.45 | 0.57 | 0.55 | 0.55 | 0.45 | 0.45 | 0.56 | 0.48 | 0.54 | 0.54 | 0.55 | 0.52 | 0.56 | 0.77 |
| FDA | 0.60 | 0.52 | 0.65 | 0.62 | 0.46 | 0.61 | 0.60 | 0.64 | 0.62 | 0.52 | 0.68 | 0.59 | 0.65 | 0.67 | 0.70 | 0.58 | 0.78 | 0.67 | 0.74 | 0.79 |
| SVM | 0.55 | 0.58 | 0.59 | 0.59 | 0.62 | 0.62 | 0.56 | 0.55 | 0.61 | 0.64 | 0.57 | 0.64 | 0.57 | 0.60 | 0.57 | 0.62 | 0.61 | 0.61 | 0.64 | 0.68 |
| SVME | 0.60 | 0.57 | 0.55 | 0.50 | 0.59 | 0.54 | 0.50 | 0.56 | 0.58 | 0.58 | 0.54 | 0.59 | 0.52 | 0.55 | 0.56 | 0.61 | 0.60 | 0.56 | 0.67 | 0.63 |
| BIOCLIM | 0.57 | 0.64 | 0.57 | 0.57 | 0.62 | 0.54 | 0.67 | 0.62 | 0.64 | 0.57 | 0.68 | 0.68 | 0.68 | 0.59 | 0.56 | 0.68 | 0.66 | 0.69 | 0.64 | 0.65 |
| DOMAIN | 0.58 | 0.65 | 0.61 | 0.56 | 0.72 | 0.58 | 0.56 | 0.59 | 0.63 | 0.62 | 0.67 | 0.65 | 0.63 | 0.62 | 0.59 | 0.60 | 0.69 | 0.74 | 0.65 | 0.66 |
| Mahal | 0.62 | 0.68 | 0.57 | 0.70 | 0.61 | 0.51 | 0.72 | 0.62 | 0.66 | 0.62 | 0.51 | 0.62 | 0.62 | 0.67 | 0.66 | 0.77 | 0.52 | 0.64 | 0.63 | 0.68 |
| Ensemble | 0.61 | 0.61 | 0.62 | 0.63 | 0.63 | 0.63 | 0.64 | 0.65 | 0.65 | 0.65 | 0.66 | 0.67 | 0.68 | 0.69 | 0.69 | 0.72 | 0.73 | 0.74 | 0.75 | 0.80 |

* Value of this calibration is used for further analysis
