# Supplementary material for: Suitability Analysis and Projected Climate Change Impact on Banana and Coffee Production Zones in Nepal
Source: PLoS One. 2016 Sep 30;11(9):e0163916. doi: 10.1371/journal.pone.0163916 (PMC5045210; doi:10.1371/journal.pone.0163916)
Supplement: S7 Table — (DOC) [file pone.0163916.s009.doc]

**S7 Table. cAUC and weight**

**For banana**

| Model | Weight | | |
| --- | --- | --- | --- |
|  | cAUC | ENSEMBLE.min=0.5 | Input weight* = 0.05 |
| MAXENT | 0.7246094 | 0.0582 | 0.0673 |
| GBM | 0.7119141 | 0.0572 | 0.0661 |
| GBMSTEP | 0.6669922 | 0.0536 | 0.062 |
| RF | 0.6923828 | 0.0556 | 0.0643 |
| GLM | 0.7597656 | 0.061 | 0.0705 |
| GLMSTEP | 0.7626953 | 0.0612 | 0.0708 |
| GAM | 0.78125 | 0.0627 | 0.0725 |
| GAMSTEP | 0.7792969 | 0.0626 | 0.0724 |
| MGCV | 0.7705078 | 0.0619 | 0.0716 |
| EARTH | 0.7089844 | 0.0569 | 0.0658 |
| RPART | 0.6865234 | 0.0551 | 0.0637 |
| NNET | 0.550293 | 0.0442 | 0 |
| FDA | 0.6875 | 0.0552 | 0.0638 |
| SVM | 0.6787109 | 0.0545 | 0.063 |
| SVME | 0.7382812 | 0.0593 | 0.0686 |
| BIOCLIM | 0.5717773 | 0.0459 | 0 |
| DOMAIN | 0.6230469 | 0.05 | 0.0578 |
| MAHAL | 0.5605469 | 0.045 | 0 |

* Weight based on which ensemble model generated

**For coffee**

| Model | Weight | | |
| --- | --- | --- | --- |
|  | AUC | ENSEMBLE.min=0.5 | input weight* = 0.05 |
| MAXENT | 0.806213 | 0.0615 | 0.0717 |
| GBM | 0.8091716 | 0.0617 | 0.072 |
| GBMSTEP | 0.7840237 | 0.0598 | 0.0697 |
| RF | 0.7056213 | 0.0538 | 0.0627 |
| GLM | 0.7514793 | 0.0573 | 0.0668 |
| GLMSTEP | 0.795858 | 0.0607 | 0.0708 |
| GAM | 0.7085799 | 0.0541 | 0.0631 |
| GAMSTEP | 0.739645 | 0.0564 | 0.0658 |
| MGCV | 0.7647929 | 0.0583 | 0.068 |
| EARTH | 0.7618343 | 0.0581 | 0.0678 |
| RPART | 0.5821006 | 0.0444 | 0 |
| NNET | 0.7721893 | 0.0589 | 0.0687 |
| FDA | 0.7928994 | 0.0605 | 0.0706 |
| SVM | 0.6863905 | 0.0524 | 0.0611 |
| SVME | 0.6316568 | 0.0482 | 0 |
| BIOCLIM | 0.6545858 | 0.0499 | 0 |
| DOMAIN | 0.670858 | 0.0512 | 0.0597 |
| MAHAL | 0.6908284 | 0.0527 | 0.0615 |

* Weight based on which ensemble model generated
